# Luminol and the postmortem interval estimation — influence of taphonomic factors

**DOI:** 10.1007/s00414-023-03132-9

**Published:** 2023-11-24

**Authors:** Catarina Ermida, Eugénia Cunha, Maria Teresa Ferreira

**Affiliations:** 1 Department of Life Sciences, Centre for Functional Ecology (CFE), Laboratory of Forensic Anthropology, University of Coimbra, Calçada Martim de Freitas, 3000-456 Coimbra, Portugal; 2 National Institute of Legal Medicine and Forensic Sciences (INMLCF), South Branch, Rua Manuel Bento de Sousa 3, 1150-334 Lisbon, Portugal

**Keywords:** Postmortem interval, Luminol, Taphonomy, Influencing factors, Forensic anthropology

## Abstract

The estimation of the postmortem interval (PMI) is one of the key challenges for forensic anthropologists. Although there are several methods referenced for this purpose, none is sufficiently effective. One of the main reasons justifying the complexity of this task is the influence of several taphonomic factors.

The study of the Luminol technique has stood out as a promising method for estimating PMI, complementing the existing methods, since it is an economic, easy and reproducible method that operates as a presumptive test. However, it is not known which taphonomic factors can influence the results obtained by this technique.

The aim of this study is to test the influence of taphonomic factors, such as temperature, humidity, soil type and pH, on the estimation of the PMI by the Luminol technique.

In order to test the influence of the referred factors, a sample consisting of 30 clavicles, with known and similar PMI, collected from autopsies, was distributed as evenly as possible by six vases and buried with different decomposition conditions for a period of 12 months. After the exhumation and sample preparation, the Luminol technique was applied.

It was possible to clearly observe differences in the results. Thus, according to our research, it is possible to conclude that the results obtained by the application of Luminol are influenced by taphonomic factors. Therefore, the context in which a body is found should always be considered for applying this technique.

## Introduction

The estimation of the postmortem interval imposes one of the main challenges in forensic sciences, corresponding to the time interval since death until the discovery of the body. This parameter is essential for the verification of the forensic relevance of the human remains. However, its accuracy is strongly related to the time elapsed since death, as the longer the PMI the more difficult an accurate estimation [1–4].

Although the last decades have reflected an increase in the number of studies aimed at finding appropriate methodologies for PMI estimation, the attempt to date skeletonized remains still proves to be a challenge for forensic anthropologists [5, 6]. The complexity of this task is essentially related to the number of factors that can influence the natural evolution of changes that occur after death [7]. Yet, the majority of published studies do not reflect the influence of these factors, leading to biased results. Thus, a holistic approach, where all the pieces of evidence are precious indicators, is fundamental for a reliable time since death estimation [4]. However, numerous factors contribute to the complexity of this type of research. These include intricate logistical conditions for the experimental process, such as stringent security procedures due to the involvement of human samples. Additionally, the challenges in gathering human samples necessitate thorough ethical approvals, which frequently entail complex and time-consuming bureaucratic procedures that can even be unattainable in certain legal contexts.

According to Vass [8], the decomposition process primarily depends on temperature and moisture, to a lesser extent. In 2011, Vass complements its previous study, stating that temperature, moisture, pH, and partial pressure of oxygen constitute the four generally recognized factors that influence the rate of the decomposition process [9]. Temperature is considered among the scientific community as the most important factor affecting the decaying process [10], since it controls humidity extent, conservative processes development such as adipocere and mummification, insects and scavenging activity, or bacterial growth [11]. On the other hand, it can be influenced by the burial depth, air movement, time of the year, or even the presence of vegetation or clothing [9]. In general, an increase in temperature results in an accelerated progress of a cadaver through the decomposition stages [12–14]. This can be explained since it is naturally associated with an increase in biological activity [15]. Water presence/absence in the environment surrounding the cadaver can also affect its degradation, as it acts as a diluent, as a source of H^+^ for biochemical reactions and support for microbial activity, as a solvent for polar molecules, or as a buffer moderating the effects of pH changes. It can also stabilize temperatures, and it is positively related to insect activity [9, 12, 16]. Without water, the biological processes cease. Although, according to Campobasso et al. [17], decomposition can be slowed by water-saturating environments, limited by the lack of oxygen. At the present, the influence of the soil pH on the body’s decay is not clear, although it is known that it is less influential than other parameters like temperature or moisture. Even though, it can be inferred that acidic soils may slow the decomposition process down, due to plants’ great production of tannins, resulting in an inhibition of bacterial growth [10]. The last factor widely associated with decomposition referred by Vass [9] is the partial pressure of oxygen. This parameter is extensively influenced by moisture, altitude, and, most of all, burial depth. Low oxygen content in the burial environment tends to slow decomposition since it delays the oxidative processes [18]. Several other factors can affect the breakdown of a cadaver, including the burial depth and cadaver’s deposition (indoor vs. outdoor), the presence of vegetation, the physical condition of the body (injuries, diseases, or body fat/mass), and the presence of clothes or insect or scavenging activity [9]. Overall, body decomposition is a well-known process; however, the timing of death remains extremely complex. The reason behind this problem lies in the numerous factors affecting this process and their mutual influence over each other.

In recent decades, the Luminol technique has been studied to estimate PMI in skeletonized remains as a presumptive test, since it is considered a simple and inexpensive technique, able to be applied and interpreted by the naked eye [19, 20]. With the application of Luminol (a cyclic acyl hydrazide) on haemoglobin traces within the bone tissue, a chemiluminescence reaction occurs, corresponding to the emission of light ranging between violet and a blue background derived from the chemical reaction [21–24]. For this reaction to take place, a redox catalyst is required, the iron ions contained in the haemoglobin composition, present in bones [25, 26]. Over time, the amount of haemoglobin in skeletonized remains decreases [22, 27, 28]. Consequently, it is expected a decrease in the intensity of the chemiluminescence reaction with the increasing postmortem interval [20, 28, 29].

This work aims to verify the influence of temperature, humidity, type of soil, and pH, on the PMI estimation through Luminol’s technique on buried bones.

## Material and methods

For our study, 30 clavicles of adult individuals (age-at-death between 25 and 86 years old) of both sexes, without visible or known bone pathology or antemortem/perimortem trauma (Table 1), were extracted during autopsies from the Portuguese Legal Medicine and Forensic Sciences National Institute, Centre Branch (INMLCF, IP), after approval from the Ethics Commission (CE-010/2018) and consulting of RENNDA – Portuguese National Register of Non-Donors, along a 5 months’ period. All the individuals had similar and known PMI (between 1241 and 1090 days).

**Table 1:** Sample data (PMI, sex, and age-at-death per individual)

Sample	PMI (days)	Sex	Age-at-death
1	1241	M	80
2	1241	F	76
3	1240	M	79
4	1239	F	59
5	1229	M	58
6	1220	M	84
7	1220	M	55
8	1220	M	71
9	1218	M	65
10	1216	M	72
11	1216	M	80
12	1212	M	25
13	1205	F	61
14	1200	F	40
15	1199	F	28
16	1198	M	86
17	1198	M	63
18	1192	M	41
19	1187	M	34
20	1177	M	59
21	1177	M	66
22	1177	F	83
23	1175	M	70
24	1167	F	69
25	1166	M	57
26	1120	M	58
27	1110	F	76
28	1093	F	67
29	1091	M	67
30	1090	M	79

*PMI* Postmortem interval; *M* male; *F* female

**Fig. 1:**
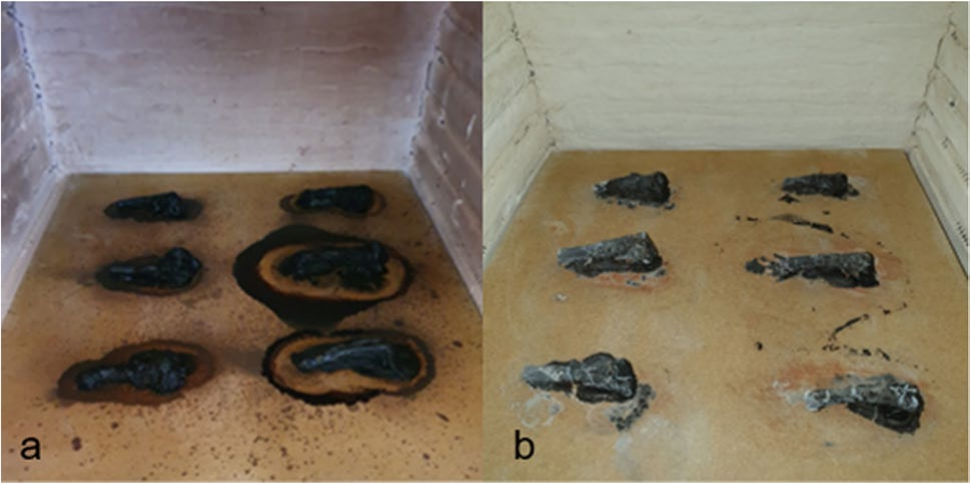
Burned clavicles in the electric muffle: **a** 400 °C for 30 min; **b** 700 °C for 60 min

The experience was conducted in the terrace of the Department of Life Sciences, University of Coimbra. To meet the mandatory conditions for the experience, the bones were preserved by freezing at 4 °C in labelled zip-lock freezer bags until the inhumation date. When the necessary conditions were met (availability of the required material and space), the bones were defrosted at room temperature and sawn in half with a power saw (the sternal end was used for this work and the acromial end was saved for later experiences). In order to test the influence of the different taphonomic factors, the sample was distributed as evenly as possible over six vases, and shallowly buried under different decomposition conditions — different soil types (sandy soil and two substrates with different pH values — Universal Substrate pH 6.9 and COMPO SANA pH 4), outdoors or inside a greenhouse, for a period of 12 months. Sex and age-at-death were also taken into consideration so that the distribution was as equitable as possible. Two clavicles per vase were burned (before the inhumation) under controlled conditions, using an electric muffle (Barracha, K-3 three-phased, 14A), one at 400° for 30 min and the other at 700° for 60 min (from room temperature until aimed maximum temperature) (Fig. 1).

**Fig. 2:**
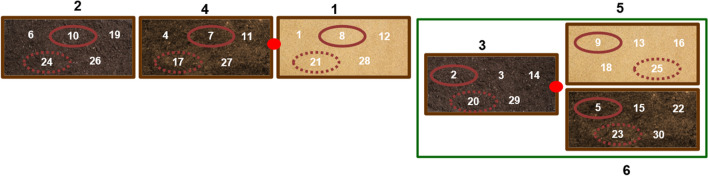
Mapping of the experience: 1 — sandy soil outdoor; 2 — COMPO SANA pH 4 outdoor; 3 — COMPO SANA pH 4 indoor; 4 — universal substrate pH 6.9 outdoor; 5 — sandy soil indoor; 6 — universal substrate pH 6.9 indoor;  — greenhouse;  — burned at 400 °C, 30 min;  — burned at 700 °C, 60 min;  — data logger

**Fig. 3:**
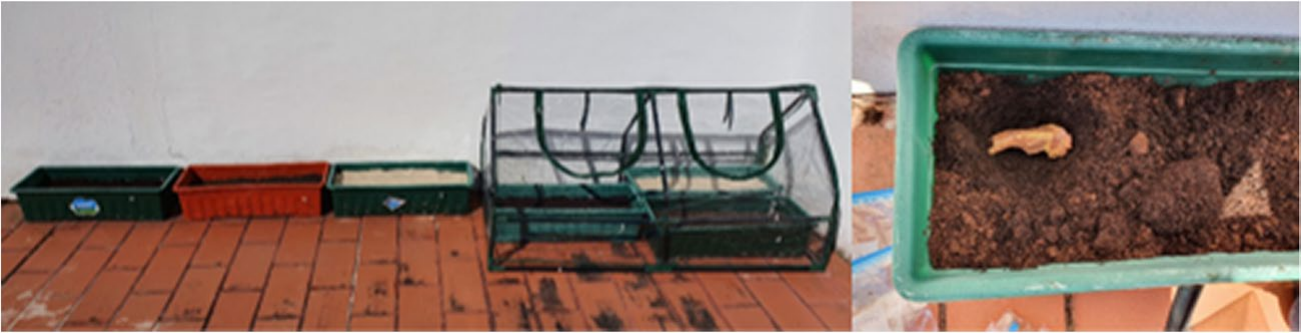
Disposition of the experience

A map of the experience was drawn (Figs. 2 and 3), with five properly identified clavicles, placed in each vase, each one in an individual hole. Temperature monitoring, both inside the greenhouse and outside, was controlled every 60 min by two Data Loggers (Embedded Data Systems Thermochron iButton DS1921G-F5#). Inside the greenhouse (indoors), the average temperature was 31.7 °C, with a maximum of 62 °C and a minimum of 20 °C. In the open air (outdoors), the average temperature was 26 °C, with a maximum temperature of 45 °C and a minimum of 17 °C. According to the Portuguese Institute of the Sea and Atmosphere, the medium temperature in Coimbra within the 12 months was 21.4 °C, with a maximum temperature of 40 °C and a minimum of 8 °C. Regarding precipitation, it fluctuated between 10 and 25 mm in the summer months and 150–200 mm in the autumn/winter months.

After 12 months, the sample was exhumed, processed, and the technique was applied. The sample treatment involved manual cleaning with sandpaper and brushes and the reduction to powder resourcing to a scalpel. For each clavicle, 50 mg of powder was collected in an Eppendorf tube for the Luminol technique. The Luminol 16 oz solution was prepared according to the directions included in the reagent. Each sample was placed on a clean white paper sheet, and Luminol was applied in spray at room temperature, in a dark room. As a positive control, a drop of blood was used, and as a negative control, a powdered bone dated from the nineteenth century. The results were examined with the naked eye by two different observers, and photographed with a Canon EOS 500D (F/5, 30 s, ISO 3200, 27 mm), with the total absence of light. The visual examination was conducted blindly, with the origin of the samples remaining undisclosed to both observers when the reaction occurred, and the results were recorded. The intensity of the reaction was measured using a 5-level scale, according to the increasing degree of chemiluminescence (see Table 2 for details).

**Table 2:** Interpretation of the 5-level scale for the chemiluminescence reaction

Result	Interpretation
Negative ( −)	No chemiluminescence. The reaction is not visible to the naked eye
Very weak positive ( +)	The reaction is barely visible to the naked eye
Weak positive (+ +)	The reaction is visible to the naked eye but with low intensity
Positive (+ + +)	The reaction is easily visible to the naked eye
Strong positive (+ + + +)	The reaction is strongly visible to the naked eye

## Results

**Fig. 4:**
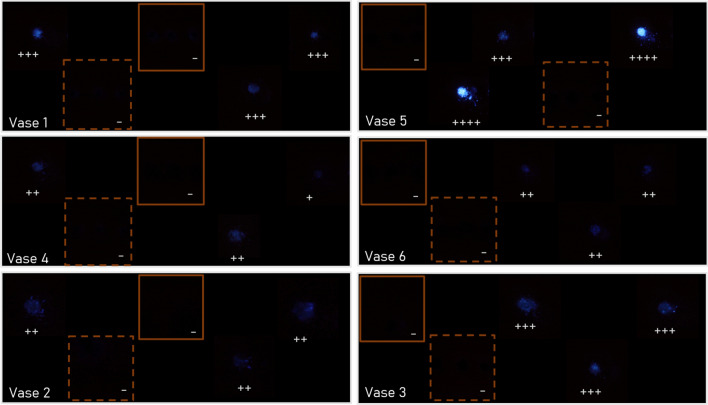
Luminol application results, according to Fig. 2 mapping: vase 1 — sandy soil outdoor; vase 2 — COMPO SANA pH 4 outdoor; vase 3 — COMPO SANA pH 4 indoor; vase 4 — universal substrate pH 6.9 outdoor; vase 5 – sandy soil indoor; vase 6 — universal substrate pH 6.9 indoor

The results obtained by Luminol’s application (Table 3 and Fig. 4) were similar for all clavicles buried in the same environmental conditions (same vase), with an exception for the burned clavicles, which did not show any chemiluminescence reaction, regardless of the deposition context. As can be seen in Table 3, there was no difference in the results achieved by the two different observers.

**Table 3:** Luminol’s technique results: X — observer 1; x — observer 2

Sample	Vase	5-level scale									
		Negative		Very weak positive		Weak positive		Positive		Strong positive	
1	1							X	x		
2	3	X	x								
3	3							X	x		
4	4					X	x				
5	6	X	x								
6	2					X	x				
7	4	X	x								
8	1	X	x								
9	5	X	x								
10	2	X	x								
11	4			X	x						
12	1							X	x		
13	5							X	x		
14	3							X	x		
15	6					X	x				
16	5									X	x
17	4	X	x								
18	5									X	x
19	2					X	x				
20	3	X	x								
21	1	X	x								
22	6					X	x				
23	6	X	x								
24	2	X	x								
25	5	X	x								
26	2					X	x				
27	4					X	x				
28	1							X	x		
29	3							X	x		
30	6					X	x

Inside the greenhouse (indoors), the highest intensity of chemiluminescence was recorded in bones buried in sandy soil, with two “strong positive” results. On the other hand, the bones buried in the substrate with pH 6.9 (“Universal substrate”) showed the least intense chemiluminescence reactions.

Outdoors, on the opposite to the greenhouse, all the vases exhibited some vegetation development. On the exhumation day, the sandy soil was completely wet, unlike the two substrates, which were dry. In the outdoor decomposition case, once again the highest intensity of chemiluminescence was verified in the clavicles buried in the sand, presenting three “positive” results, and the least intense reactions corresponded to the clavicles buried in the “Universal substrate”.

In regard to the differences in the results between the bones decomposing indoors (greenhouse) versus outdoors, a higher intensity of chemiluminescence was recorded in the bones buried inside the greenhouse.

## Discussion

Concerning the substrates (COMPO SANA and universal substrate), the clavicles buried in the COMPO SANA substrate (pH 4) revealed higher intense chemiluminescence reactions, both indoors and outdoors. As described by Vass [9], soil pH can influence intracellular chemical reactions and enzymes’ catalytic ability, affecting the microbial flora. Although it is not completely consensual through the scientific community, acidic pH seems to delay decomposition, as it inhibits bacterial growth, explaining the results obtained [10, 18]. Also, plants developed in acid soils seem to produce more tannins, which can outcome in a decrease in microbial activity [30].

In the sandy soil–buried clavicles case, few studies aimed to investigate this type of decomposition context. In this study, the bones buried in the sandy soils presented the highest intensity of chemiluminescence, both inside the greenhouse and in the open air. A study conducted by Guareschi and team [31], in a cemetery in Parma (Italy) presented a higher number of skeletonized remains in silty soils than in sandy soils, which supports our results. As one knows, soil texture is a fundamental parameter affecting the rate of decomposition, as it can influence water permeability and oxygen availability [30, 32], affecting the metabolism of microorganisms. Despite sandy soils having a certain degree of water permeability, due to their particle size, waterlogging can retard the decomposition process, since the persistent water produces a buffered environment, slowing the rate of decomposition [33, 34] As mentioned in the results section, vase no. 1 was waterlogged at the exhumation date, delaying the decomposition process, and explaining the obtained results.

According to Ceciliason et al. [11], the decomposition process requires moisture and moderate temperatures. The combination of a sandy soil with a low water content commonly promotes desiccation, generally assigned to the diffusion of gases through the soil matrix [35–37] (Inside the greenhouse, the “strong positive” chemiluminescence reactions in vase no. 5 can be explained by the high temperatures and total absence of water, which desiccated the remains, inhibiting the decomposition and resulting in its natural preservation.

Regarding the differences in the results between the clavicles buried inside the greenhouse or in the open air, the indoor decomposing bones showed higher intensity reactions than the outdoor ones. It is generally accepted that higher temperatures fasten decomposition. So, according to this parameter, our results go against what was expected. On the other hand, indoor decomposition tends to be slower than outdoor, due to the lack of humidity variations and access by scavengers and insects [11], which can explain our obtained results. As mentioned before, the complexity of PMI estimation relates to taphonomic factors, and how they can influence each other. In the greenhouse, temperatures were higher; however, the clavicles were protected from the action of rainwater, fauna, and flora, which lead to a slower decomposition [9]. All the outdoor vases presented vegetation development, in opposition to the greenhouse vases. According to Mant [38], the decomposition process tends to fasten when plants surround the remains, as additional bacteria and a layer of air between the remains and the soil are introduced into the burial [10].

Burned clavicles showed no chemiluminescence reaction, regardless of the decomposition context. These results can be explained by the destruction of the organic matter, and consequently the haemoglobin, by the burning in the electric muffle [39, 40].

The similar results obtained by the two different observers confirm the high interobserver agreement of this technique, reinforcing its value as a presumptive test for PMI estimation.

Our work was aimed at accessing the influence of temperature, humidity, soil type, and pH on time since death estimation through Luminol’s chemiluminescence technique in buried bones. To better understand the impact of these factors, we followed the methodological principles of taphonomy, designing an experiment with as few remaining variables as possible. However, in the practical realm of forensic science, in a real case, it is advisable to employ a combination of methods due to the complex nature of the decomposition process, in order to achieve a more reliable PMI estimation. The selection of the used methods depends upon the unique aspects of each case and the resources at the disposal of forensic experts, keeping in mind the constraints associated with each method. It is fundamental to acknowledge the importance of adopting a multidisciplinary approach, as the decomposition process can significantly fluctuate, influenced by environmental, individual, and anthropic factors [1, 4, 41]. According to the flow chart to be followed in case of late PMI estimation, described by Franceschetti et al. [41], the luminol technique can be used both when it is necessary to establish the forensic interest of the human remains, or when its forensic interest was previously established. Though, through our obtained results, this evaluation must be conducted carefully, considering the surrounding environment that influences the decomposition process.

The small size of the sample used should also be addressed. In our work it was not possible to increase its number due to complex logistic conditions, though, in future research a larger sample would yield more robust results, especially considering it is a subjective approach primarily involving the descriptive analysis of the results.

Remains of soft tissues in the bones after their extraction during the autopsy should also be a factor to consider, since it cannot faithfully replicate the decomposition of an entire body. Still, it is important to acknowledge the challenges associated with gathering human samples for the experimental process, making the use of an entire body impossible in our context. Nevertheless, replicating our study in a setting where the use of whole cadavers is feasible would be of great value.

When the inhumation of the clavicles occurred, the donors were already dead for a certain amount of time (the clavicles were preserved by freezing). Ideally, to test the effect of the influencing factors on PMI estimation as accurately as possible, the experiment should have been conducted using freshly collected bones extracted during autopsy. However, this was not feasible, as the experiment required all the mandatory conditions to be met, including the simultaneous burial of all the clavicles and the availability of a suitable area and the required material.

## Conclusion

According to our investigation, it is possible to conclude that the results obtained for PMI estimation through the Luminol technique are influenced by taphonomic factors such as temperature, humidity, pH, or type of soil. Therefore, the context in which a body is found must always be taken into consideration when applying this technique. Also, it is important to take into consideration that this technique is mainly used as a presumptive test, and the obtained results need further confirmation from a more robust method, such as radiocarbon analyses.

As future perspectives, it should be pointed out that the quantitative evaluation of the intensity of the chemiluminescence reaction would be relevant, especially for larger samples, for a less subjective comparison of the results, as mentioned by Sarabia et al. [24], as well as testing the influence of other taphonomic factors like the buried depth and the oxygen partial pressure.

This type of experimental approach should be developed in all the methods aiming to estimate postmortem interval, determining whether its results could or not be affected by influencing factors.

## Data Availability

All data generated or analysed during this study are included in this published article.
